# Supplementary material for: Arabidopsis P4 ATPase-mediated cell detoxification confers resistance to Fusarium graminearum and Verticillium dahliae
Source: Nat Commun. 2021 Nov 5;12:6426. doi: 10.1038/s41467-021-26727-5 (PMC8571369; doi:10.1038/s41467-021-26727-5)
Supplement: Supplementary file 1 — Supplementary Information [file 41467_2021_26727_MOESM1_ESM.docx]

***Arabidopsis* P4 ATPase-mediated cell detoxification confers resistance to *Fusarium graminearum* and *Verticillium*** ***dahliae*.**

Wang and Li et al., Supplementary information.


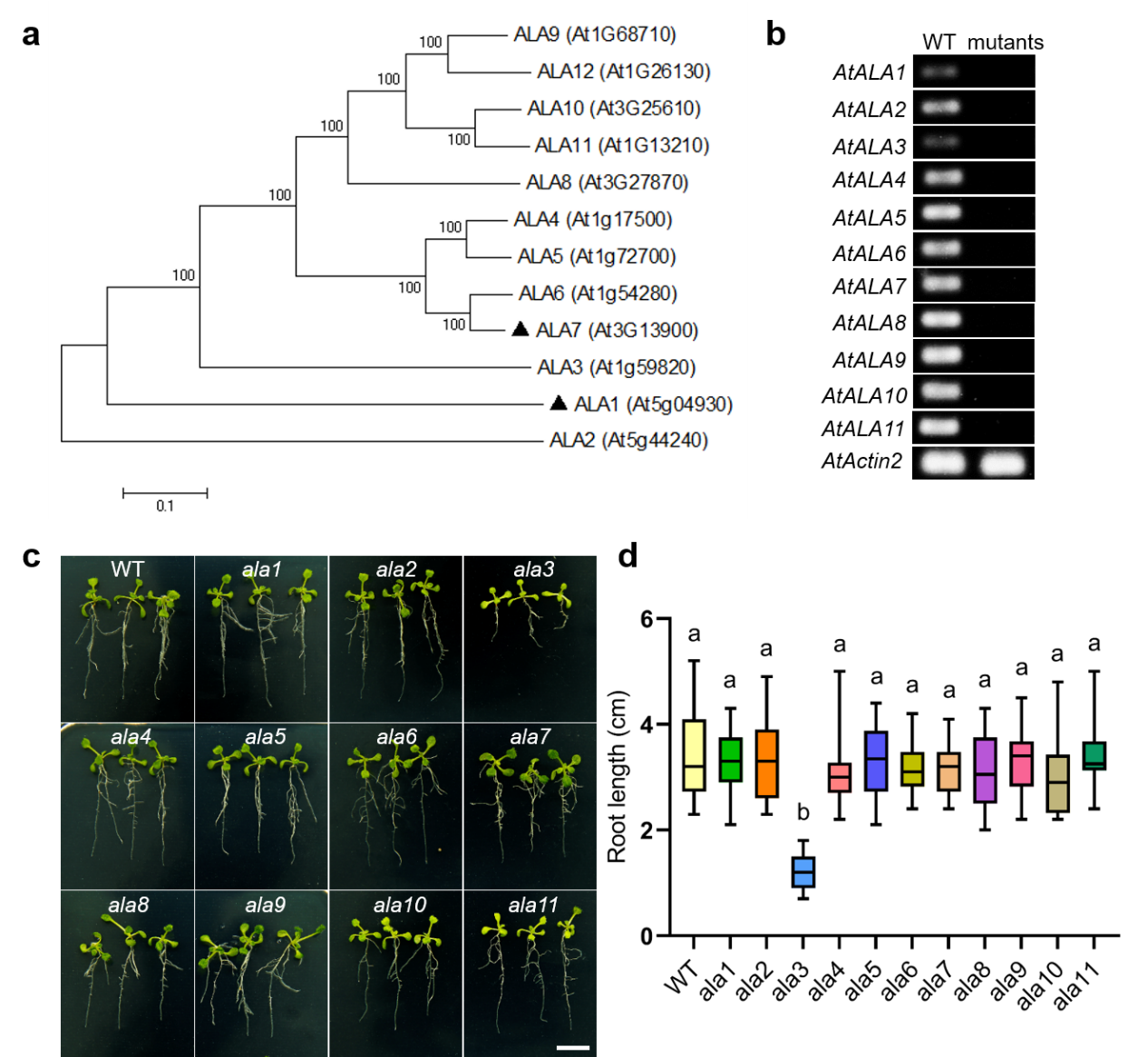


**Supplementary Figure 1. Phylogenetic analysis and phenotypic characterization** **of *Arabidopsis* P4-ATPases mutants. a** Phylogenetic analysis of the P4-ATPases protein family in *Arabidopsis*. **b** Semi-quantitative analysis showed that P4-ATPase genes (*AtALA1*-*AtALA11*) had been knocked-out in the mutants (*ala1*-*ala11*), respectively. The cDNA templates were amplified by 28 PCR cycles. The experiment was repeated three times independently with similar results. **c** Phonotypes of *P4-ATPases* mutants growing on MS medium for 10 d. WT, *Arabidopsis* wild-type (Col-0). *ala1-ala11*, AtALAs loss-of-function mutants. Scale bar, 1 cm. **d** Root length of wild-type and *AtALAs* mutants. Data are shown with box-and-whisker plots of three replicates (8 plants each). Different letters represent significant differences at P < 0.05 by one-way ANOVA with a Tukey multiple comparisons test. Box-and-whisker plots show the medians (horizontal lines), upper and lower quartiles (box edges) and 1.5× the interquartile range (whiskers).


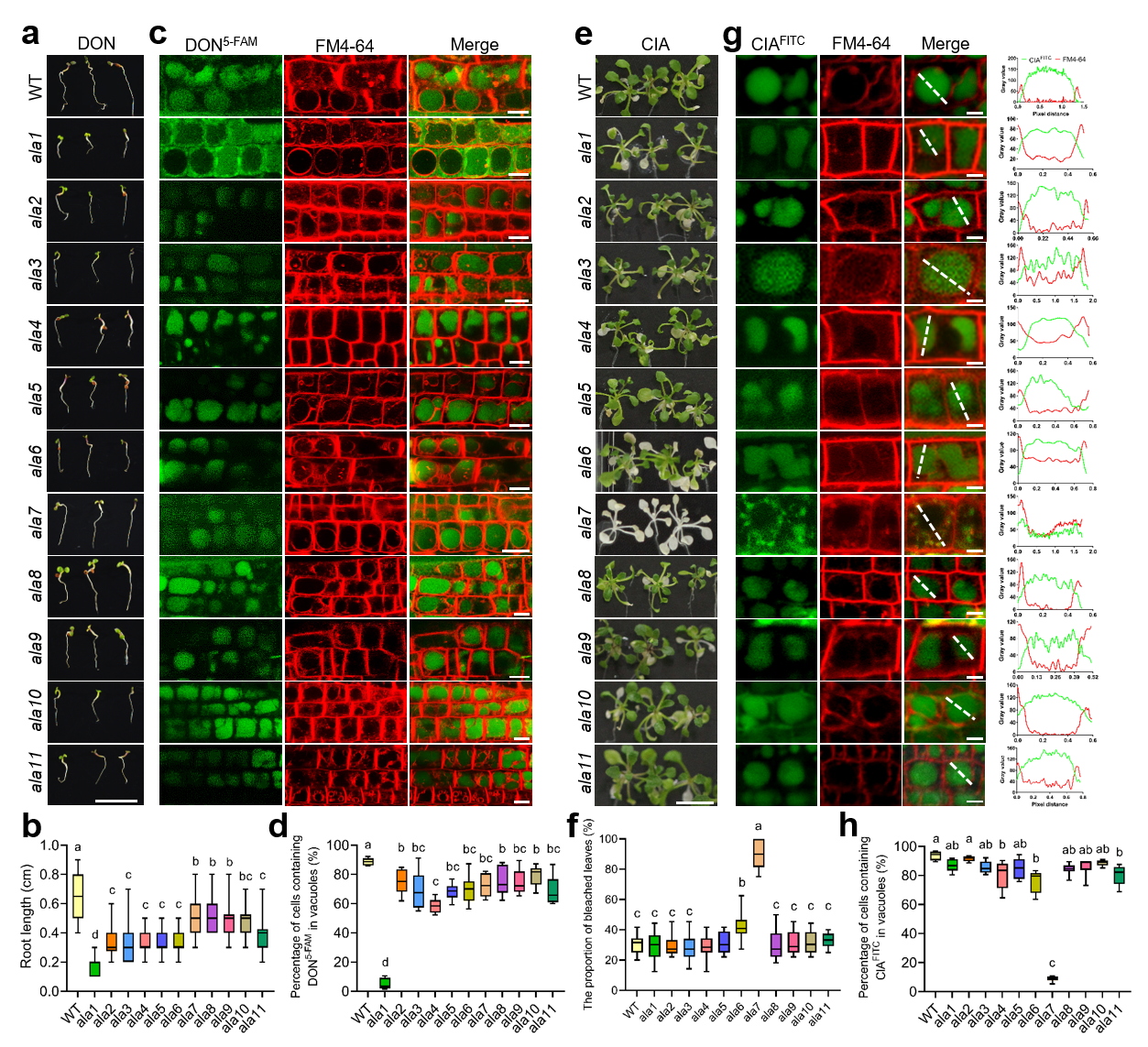


**Supplementary Figure 2. AtALA1 and AtALA7 are responsible for the accumulation of DON^5-FAM^ and CIA^FITC^ in vacuoles, respectively. a** DON tolerance assay on *Arabidopsis* P4-ATPases mutants (*ala1*-*ala11*). Two-day-old wild-type and P4-ATPases mutants (*ala1*-*ala11*) seedlings were treated with DON (1 μg·mL^-1^) for 5 d. Scale bar, 0.5 cm. **b** Root length of wild-type and P4-ATPases mutants (*ala1*-*ala11*) with DON treatment. The results are shown with box-and-whisker plots of three replicates (10 plants each). **c** Distribution of DON^5-FAM^ in root cells of wild-type and P4-ATPases mutants (*ala1*-*ala11*). Seedlings were treated with DON^5-FAM^ (9 μg·mL^-1^) and FM4-64 (8 μM) for 12 h. Scale bar, 10 μm. **d** Percentage of cells containing DON^5-FAM^ in their vacuoles, as shown with dot plots (*n =* 6 roots). **e** CIA tolerance assay of *Arabidopsis* P4-ATPases mutants (*ala1*-*ala11*). Six-day-old seedlings were transferred to MS media containing CIA (100 μg·mL^-1^) for 7 d. Scale bar, 1 cm. **f** Proportion of bleached leaves suffering from CIA. Data are shown as box-and-whisker plots of three replicates (6 plants each). **g** Distribution and fluorescence density profile of CIA^FITC^ in root cells of seedlings of wild-type and mutants (*ala1-ala11*). Membrane is stained by FM4-64 (8 μM). Scale bar, 5 μm. Measurement of fluorescence density profiles of CIA^FITC^ and FM4-64 along the white dotted lines using ImageJ. **h** Percentage of cells containing CIA^FITC^ in their vacuoles, as shown with dot plots (*n =* 7 roots). Different letters in **b**, **d**, **f** and **h** represent significant differences at P < 0.05 by one-way ANOVA with a Tukey multiple comparisons test. Box-and-whisker plots show the medians (horizontal lines), upper and lower quartiles (box edges) and 1.5× the interquartile range (whiskers).

**
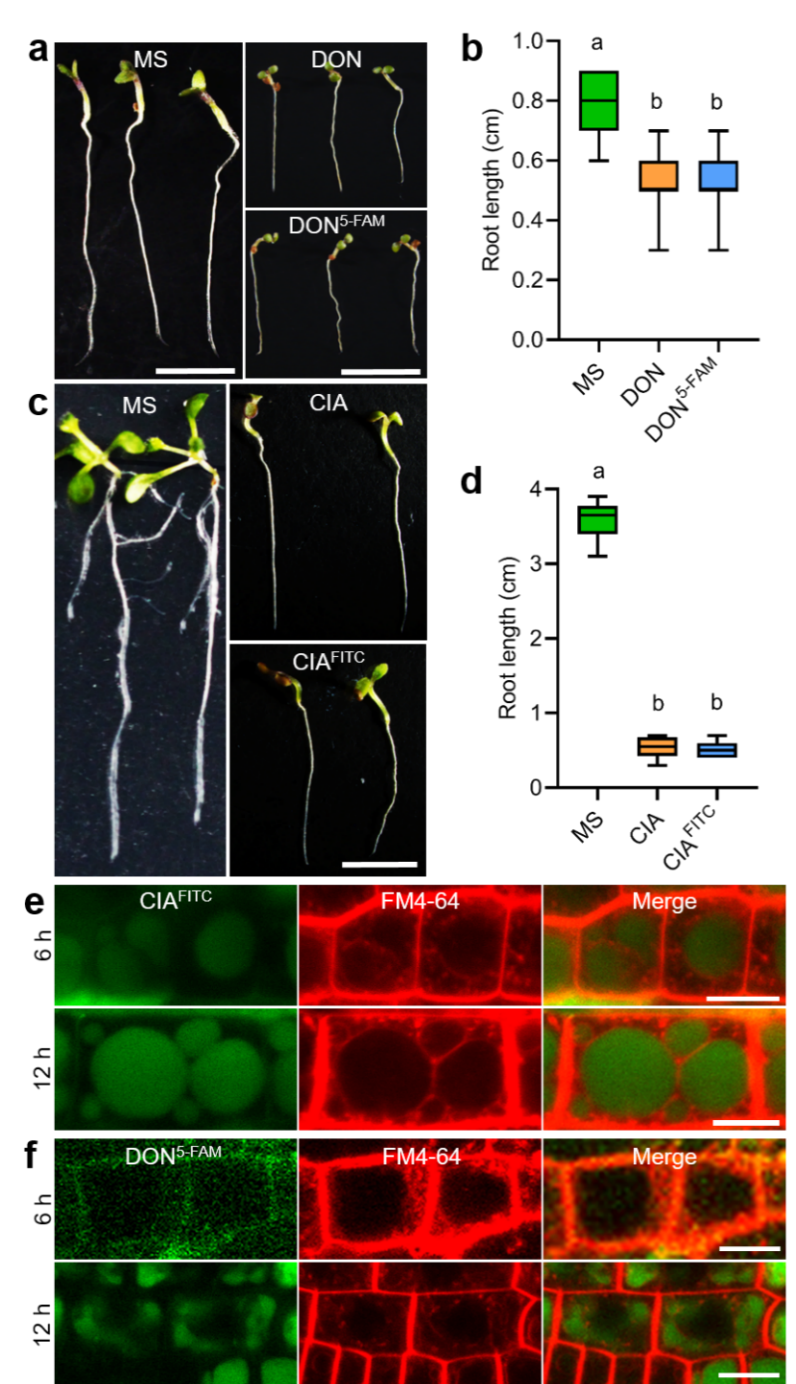
**

**Supplementary Figure 3. Tolerance assay and vacuole accumulation of DON^5-FAM^ and** **CIA^FITC^ in *Arabidopsis*. a** Tolerance assay of wild-type seedlings with the treatment of DON and DON^5-FAM^. The seedlings were exposed to DON (1 μg·mL^-1^) and DON^5-FAM^ (1 μg·mL^-1^) for 5 d. Scale bar, 5 mm. **b** The length of roots of DON- and DON^5-FAM^-treated seedlings. Data are shown with box-and-whisker plots of three replicates (10 plants each). **c** Tolerance assay of wild-type seedlings with the treatment of CIA and CIA^FITC^. The seedlings were exposed to CIA (100 μg·mL^-1^) and CIA^FITC^ (100 μg·mL^-1^) for 7 d. Scale bar, 0.3 cm. **d** Root length of CIA- and CIA^FITC^-treated seedling. The results are shown with box-and-whisker plots of three replicates (8 plants each). Different letters in **b** and **d** represent significant differences at P < 0.05 by one-way ANOVA with a Tukey multiple comparisons test. Box-and-whisker plots show the medians (horizontal lines), upper and lower quartiles (box edges) and 1.5× the interquartile range (whiskers). **e** Distribution of CIA^FITC^ in root cells of wild-type seedlings. The seedlings were treated with CIA^FITC^ (4 μg·mL^-1^) and FM4-64 (8 μM), and the fluorescence signals were observed at 6 h and 12 h, respectively. **f** Distribution of DON^5-FAM^ in root cells of wild-type seedlings. The seedlings were treated with DON^5-FAM^ (4 μg·mL^-1^) and FM4-64 (8 μM), and observed them at 6 h and 12 h, respectively. Scale bar, 10 μm. The experiments in **e** and **f** were repeated three times independently with similar results.


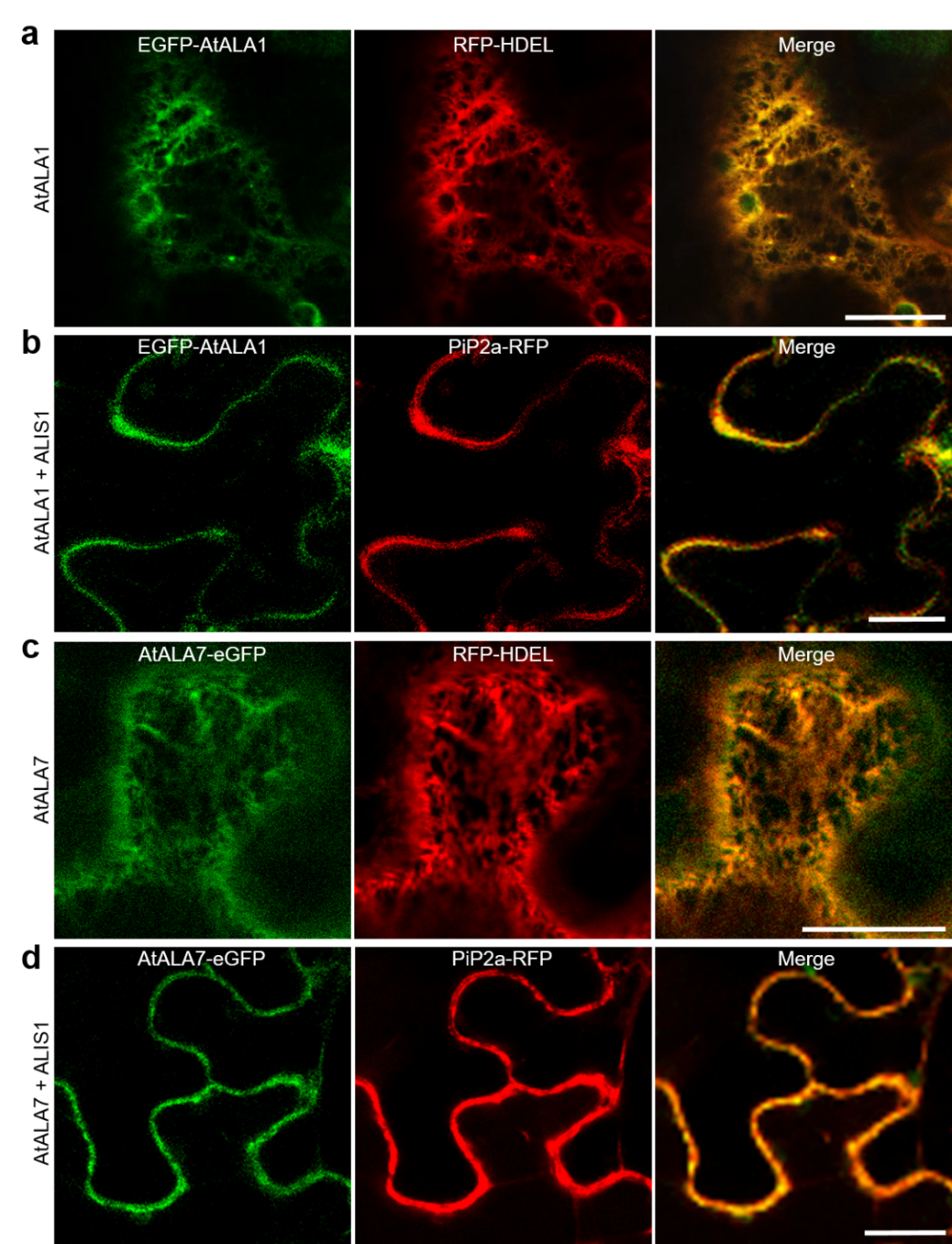


**Supplementary Figure 4. AtALA1/AtALA7 are localized to the PM, which need the help of β-subunit (ALIS1) to exit ER. a** AtALA1 colocalized with ER marker RFP-HDEL in absence of β-subunit ALIS1. AtALA1, *proAtALA1::*EGFP-AtALA1 and ER marker RFP-HDEL were transiently coexpressed in epidermal cells of *Nicotiana benthamiana* without ALIS1. **b** AtALA1 colocalized with PM marker PiP2a-RFP in presence of ALIS1. AtALA1 + ALIS1, *proAtALA1::EGFP-AtALA1*-*Nos-S7::ALIS1-Trbc* cassette and PM marker PiP2a-RFP fusion gene were transiently coexpressed in epidermal cells of *N. benthamiana*. S7, the constitutive S7 promoter. **c** AtALA7 colocalized with ER marker RFP-HDEL in absence of β-subunit ALIS1. AtALA7, *proAtALA7::*AtALA7-eGFP and ER marker RFP-HDEL were coexpressed in epidermal cells of *N. benthamiana* without ALIS1. **d** AtALA7 colocalized with PM marker PiP2a-RFP in presence of ALIS1. AtALA7 + ALIS1, *proAtALA7::AtALA7-eGFP*-*Nos-S7::ALIS1-Trbc* cassettes and PM marker PiP2a-RFP fusion gene were transiently co-expressed in epidermal cells of *N. benthamiana*. Scale bar, 20 μm. The experiments in **a** - **d** were repeated three times independently with similar results.


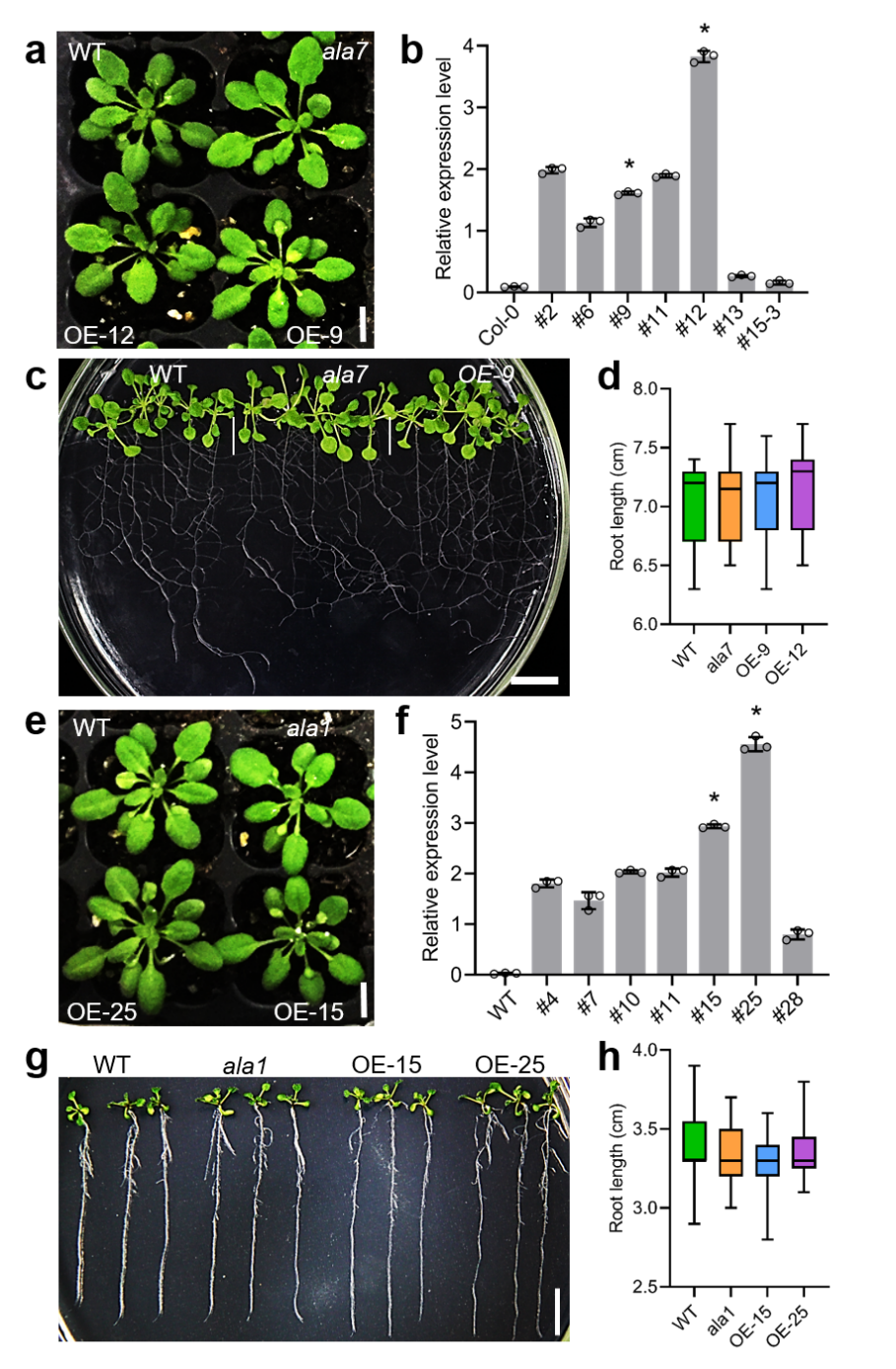


**Supplementary Figure 5.** **Neither disruption nor** **overexpression of *AtALA1* and *AtALA7* produces significant impact on *Arabidopsis* growth**. **a** The rosette leaves of wild-type, *ala7* mutant and *35S::AtALA7* transgenic lines. Scale bar, 1 cm. **b** The transcript levels of *AtALA7* in wild-type and *35S::AtALA7* transgenic *Arabidopsis* lines (n = 3 biologically independent experiments). *The line selected for further study. **c** The seedlings phenotype of wild-type, *ala7* mutant and *35S::AtALA7* transgenic lines. Scale bar, 1 cm. **d** Root length of wild-type, *ala7* mutant and *35S::AtALA7* transgenic lines. WT, wild-type; *ala7*, AtALA7 loss-of-function mutant; OE-9 and OE-12, *35S::AtALA7* overexpression *Arabidopsis* lines. Data are shown with box-and-whisker plots of three replicates (8 plants each). **e** The rosette leaves of *AtALA1* transgenic *Arabidopsis* lines. Scale bar, 1 cm. **f** The transcript levels of *AtALA1* in wild-type and *35S::AtALA1* transgenic *Arabidopsis* lines (n = 3 biologically independent experiments). *AtActin2* was used as internal standard for gene expression analysis. *The line selected for further study. **g** The seedlings phenotype of wild-type, *ala1* mutant and *AtALA1* transgenic lines. Scale bar, 1 cm. **h** Root length of wild-type, *ala1* mutant and *35S::AtALA1* transgenic lines. WT, wild-type. *ala1*, AtALA1 loss-of-function mutant. OE-15 and OE-25, *35S::AtALA1* overexpression *Arabidopsis* lines. The results are shown with box-and-whisker plots of three replicates (7 plants each). Data in **b** and **f** are presented as the means ± s.e.m. Box-and-whisker plots in **d** and **h** show the medians (horizontal lines), upper and lower quartiles (box edges) and 1.5× the interquartile range (whiskers).


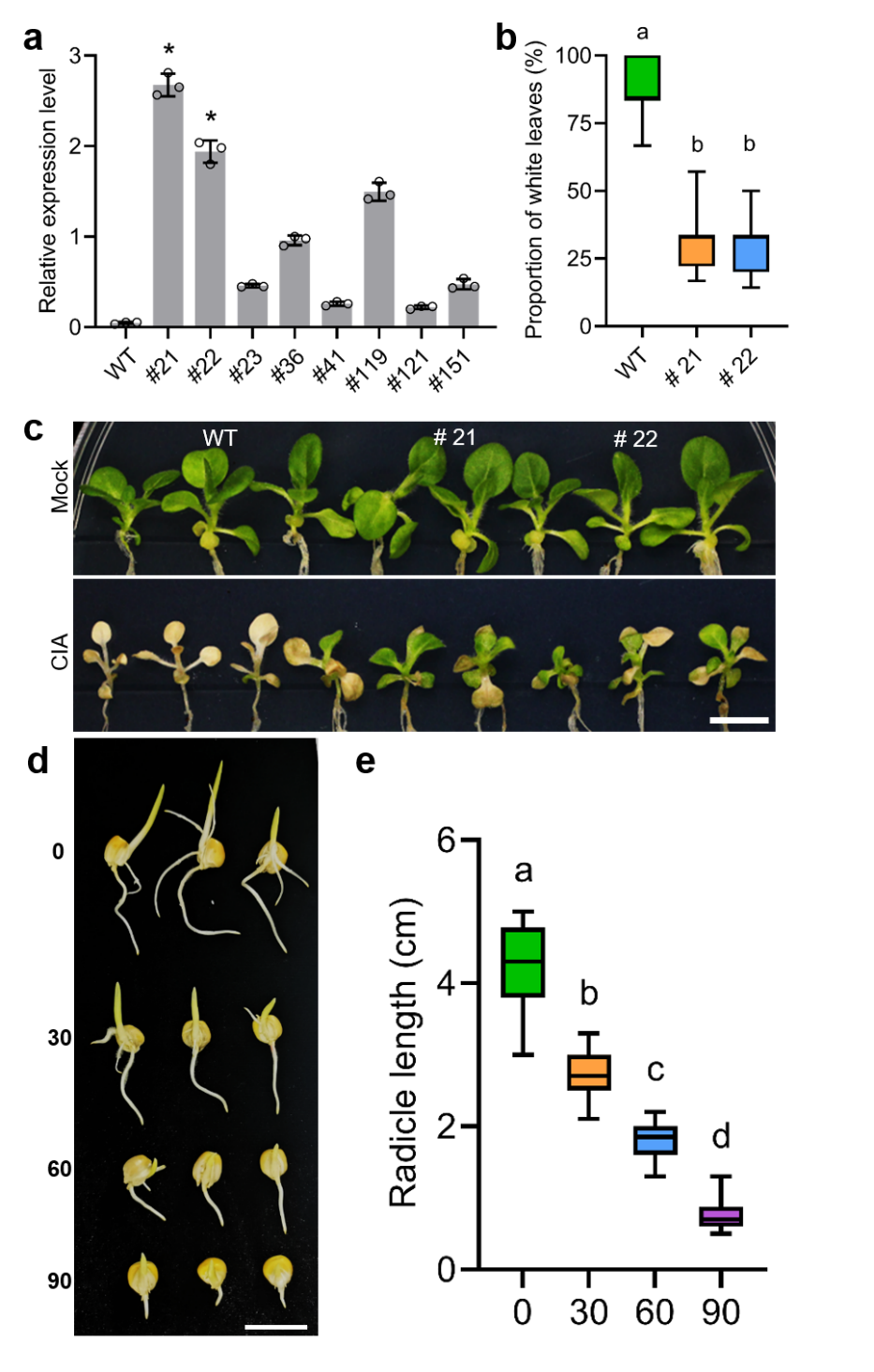


**Supplementary Figure 6. CIA tolerance assay in *35S::AtALA7* transgenic tobacco and DON tolerance assay in wild-type maize. a** Transcript levels of *AtALA7* in *35S::AtALA7* transgenic tobacco lines (n = 3 biologically independent experiments). *NtActin* was used as internal standard for gene expression analysis. *, the line selected for further study. Data are presented as the means ± s.e.m. **b** Proportion of bleached leaves of the wild-type and *AtALA7* transgenic tobacco lines (# 21 and # 22) with CIA treatment. The results are shown with box-and-whisker plots of three replicates (8 plants each). **c** Leaf phenotype of the wild-type and *AtALA7* transgenic tobacco lines (# 21 and # 22) with or without CIA treatment. The seedlings were grown on MS media containing CIA (450 μg·mL^-1^) for 3 d. Scale bar, 0.5 cm. **d** DON tolerance assay of wild-type maize seeds. Seeds (maize Hi II) were treated with different concentration of DON (0, 30, 60 and 90 μM) for 2 d. Scale bar, 2 cm. **e** Radicle elongation was inhibited with DON treatment, as shown with box-and-whisker plots of three replicates (8 plants each). Box-and-whisker plots show the medians (horizontal lines), upper and lower quartiles (box edges) and 1.5× the interquartile range (whiskers). Different letters in **b**, **e** represent significant differences at P < 0.05 by one-way ANOVA with a Tukey multiple comparisons test. Box-and-whisker plots show the medians (horizontal lines), upper and lower quartiles (box edges) and 1.5× the interquartile range (whiskers).


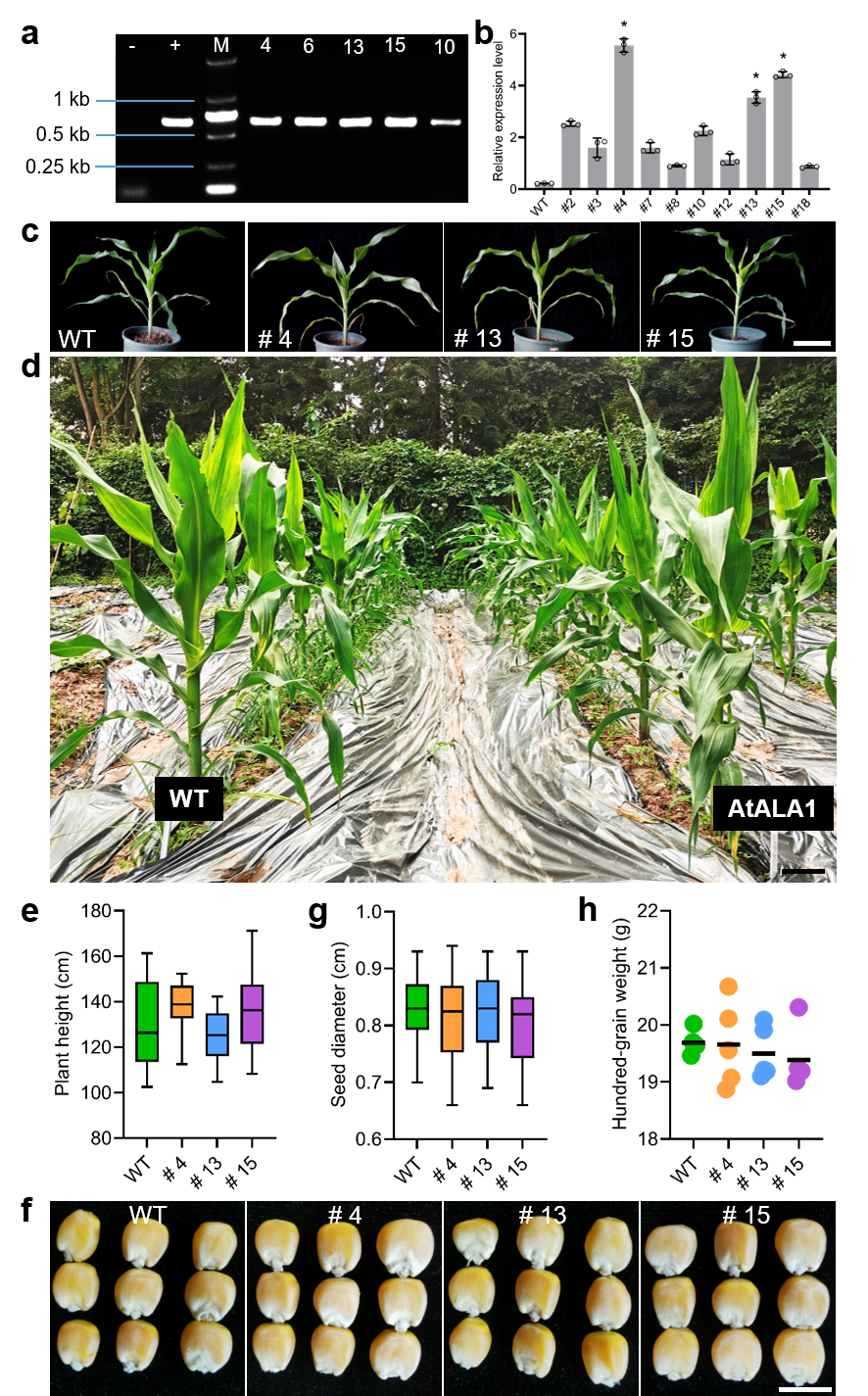


**Supplementary Figure 7. Expression of *AtALA1* does not produce significant impact on the growth and seed size of maize. a** PCR analysis of *Ubi1::AtALA1* transgenic maize. M, DNA marker DL2000. -, wild-type DNA as template. +, plasmid containing *Ubi1::AtALA1* as template. DNA samples of *Ubi1::AtALA1* transgenic maize lines 4, 6, 13, 15 and 10 as template. The experiment was repeated three times independently with similar results. **b** Transcript levels of *AtALA1* measured by qRT-PCR in *Ubi1::AtALA1* transgenic maize lines (n = 3 biologically independent experiments). *ZmEF1a* was used as internal standard for gene expression analysis. *, the line selected for further study. Data are presented as the means ± s.e.m. **c** The phenotype of *AtALA1* transgenic lines (# 4, # 13 and # 15) and wild-type (Hi II) growing in pots. Scale bar, 5 cm. **d** The phenotype of AtALA1 transgenic lines (# 4) and wild-type growing in experimental plots. Scale bar, 10 cm. **e** Plants height of wild-type and *AtALA1* transgenic maize lines. The results are shown with box-and-whisker plots (*n* = 12 plants). **f** Corn seeds of wild-type and *Ubi1::AtALA1* transgenic maize lines (# 4, # 13 and # 15). Scale bar, 1 cm. **g** Seed diameter of wild-type and *Ubi1::AtALA1* transgenic maize lines (# 4, # 13 and # 15). Data are shown with box-and-whisker plots of three replicates (each replicate contained 12 seeds derived from 3 individual plants). **h** Hundred-grain weight of wild-type and *Ubi1::AtALA1* transgenic maize lines. Data are shown with dot plots (*n =* 5 ears from individual plants). Box-and-whisker plots show the medians (horizontal lines), upper and lower quartiles (box edges) and 1.5× the interquartile range (whiskers).

**Supplementary Table 1. PCR primer sequences used in this article**

| **Primer name** | **Sequence 5'-3'** |
| --- | --- |
| LBb1.3 | ATTTTGCCGATTTCGGAAC |
| *ala1-LP* | GCCATTGGTGATGGTAATGAC |
| *ala1-RP* | ACCAGAACATCCATGTCTTGC |
| *ala1-8-LP* | CGTTTATCCCGATTTAGTAATTGTG |
| *ala1-8-RP* | CGAGCATCTTCGTCTTTGATC |
| *ala2-LP* | TACAGCAAGCTGATATTGGGG |
| *ala2-RP* | CATGCATATTTGAATGCGATG |
| \| *ala3-LP* \| \| --- \| | TTAATTTCAAGCCAAATTCGG |
| *ala3-RP* | AAGAAGCACCATTGACAGTGG |
| *ala4-LP* | TGATCCAATTAATTGAACGGG |
| *ala4-RP* | ATTCTACCTCTTGCCATTCCC |
| \| *ala5-LP* \| \| --- \| | GATCCCAAGATTCAAAAAGGG |
| *ala5-RP* | GGGAACTACACAAGCACAAGC |
| *ala6-LP* | CCTGTCAGCGCCTTTACATAG |
| *ala6-RP* | GTGTTGTGGTTTTTACTGGCC |
| *ala7-LP* | GGGGCGTCGTAGAATAAGATC |
| *ala7-RP* | GTTAACCGCTCAGGCTTATCC |
| *ala7-24-LP* | GTCTTCCACTGCAGTAGCACC |
| *ala7-24-RP* | TTGCAGATGTATGACAGCGAG |
| *ala8-LP* | GAACACTTCACCGGATCATTC |
| *ala8-RP* | TCGTAGTTGAACCTTGGGATG |
| *ala9-LP* | CACCAAAACAAAAAGCTCTGG |
| *ala9-RP* | AGTTCTCCGTACACCGTGATG |
| *ala10-LP* | ATGTGTTCGTTGGAACTTTGG |
| *ala10-RP* | ACCTTTCAACTTTCTCTCCCG |
| *ala11-LP* | ATGGCTTGCGATTAATCAATG |
| *ala11-RP* | ACTTTTGGTGTCACCGTGTTC |
| AtALA1-F (qRT-PCR) | CCGCCTCATCACGATCCAA |
| \| AtALA1-R (qRT-PCR) \| \| --- \| | CCAGCTGAACCATGACGGAT |
| AtALA7-F (qRT-PCR) | TGACCCACCTTCCAAGGACT |
| AtALA7-R (qRT-PCR) | TACCAGTGGACTGGGTCTGA |
| AtActin2-F (qRT-PCR) | GATTCAGATGCCCAGAAAGTCTTG |
| AtActin2-R (qRT-PCR) | TGGATTCCAGCAGCTTCCAT |
| ZmEF1a-F (qRT-PCR) | TGGGCCTACTGGTCTTACTACTGA |
| ZmEF1a-R (qRT-PCR) | ACATACCCACGCTTCAGATCCT |
| NtActin-F (qRT-PCR) | GGGTTTGCTGGAGATGATGCT |
| NtActin-R (qRT-PCR) | GCTTCATCACCAACATATGCAT |
| ITS1-F(qRT-PCR) | AAAGTTTTAATGGTTCGCTAAGA |
| ST-VE1-R(qRT-PCR) | CTTGGTCATTTAGAGGAAGTAA |
| Fg16N-F (qRT-PCR) | ACAGATGACAAGATTCAGGCACA |
| Fg16N-R (qRT-PCR) | TTCTTTGACATCTGTTCAACCCA |
| AtPIP2a-F (*Bam*H I) | CGGGATCCATGGCAAAGGATGTGGAAGC |
| AtPIP2a-R (+linker） | accagaaccaccaccagaaccaccGACGTTGGCAGCACTTCTGA |
| \| RFP-F (+linker） \| \| --- \| | ggtggttctggtggtggttctggtATGGCCTCCTCCGAGGACGTCAT |
| RFP-R (*EcoR* I) | GGAATTCTTAGGCGCCGGTGGAGTGGCGGCCCTCGGCGCGCTCGTACTGTT |
| γ-TIP-F(*Bam*H I) | CGGGATCCATGCCGATCAGAAACATCG |
| γ-TIP-R (+linker） | accagaaccaccaccagaaccaccGTAGTCTGTGGTTGGGAGC |
| RFP-R (*BamH* I) | CGGGATCCATGGCCTCCTCCGAGGACGTCAT |
| RFP-R (+linker) | accagaaccaccaccagaaccaccGGCGCCGGTGGAGTGGCGGCCCTCGGCGCGCTCGTACTGTT |
| RabA1d-F (+Linker) | ggtggttctggtggtggttctggtGTGTTAATGGCGGGTT |
| RabA1d-R (*Kpn* I) | GGGGTACCTTTAGGACATAAGACCAT |
| RabF2a-F (+Linker) | ggtggttctggtggtggttctggtATGGCTAGCTCTGGAAACA |
| RabF2a-R (*Kpn* I) | GGGGTACCCTAAGCACAACACGATGAACTCACT |
| AtALA1-F (*Sma I*) | TCCCCCGGGATGGATCCCAGGAAATCAATTG |
| AtALA1-R (*Sal I*) | ACGCGTCGACTCATCTCCGTGGAGGATCCTGA |
| EGFP-F (*Sma* I) | ACGGATCCGTACTAGTCCCGGGATGGTGAGCAAGGGCGAG |
| EGFP-R (+linker) | accagaaccaccaccagaaccaccTTACTTGTACAGCTCGTCCATGC |
| AtALA1-F (+linker） | ggtggttctggtggtggttctggtATGGATCCCAGGAAATCAATTGA |
| AtALA1-R (*Sal* I) | GGAATTCTGGTACCATGTCGACTCAGAAAGCAATGAAATTCT |
| Pro-ALA1-F(*Hind* Ⅲ) | acaaaggttaaattagaaaagcttTTAGTGTATGTTATTATGACGATA |
| Pro-ALA1-R(-EGFP) | TGCTCACCATGGTGGCGACTTCCCGGGTCCAGGAATTGGATTTG |
| EGFP-F(-Pro) | AATTCCTGGACCCGGGAAGTCGCCACCATGGTGAGCAA |
| AtALA1-R(-Pro) | cgggcccgggactagtacggatccTCATCTCCGTGGAGGATCCT |
| AtALA7-F(*Spe I*) | GACTAGTATGGGGCGTCGTAGAATAAGATC |
| AtALA7-R (+linker） | ACCAGAACCACCACCAGAACCACCACTATGTTGTGTGGTGGAAGTTG |
| Pro-ALA7-F(*Hind*Ⅲ) | caaaggttaaattagaaaagcttCAGGCTGCAGAAGAAAACTCTAG |
| Pro-ALA7-R(-EGFP) | TTCTACGACGCCCCATactagtAATCTCTCAAAGTTTCAGCTCAAG |
| AtALA7-F(-Pro) | CTGAAACTTTGAGAGATTactagtATGGGGCGTCGTAGAATAAGAT |
| EGFP-R(*Bam*HⅠ) | cgggcccgggactagtacggatccTTACTTGTACAGCTCGTCCATG |
| EGFP-F (+linker） | ggtggttctggtggtggttctggtATGGTGAGCAAGGGCGAG |
| EGFP-R(*Kpn* I) | GGGGTACCTTACTTGTACAGCTCGTCCATGC |
| Ubi1-F (*Sal* I) | AAGCTTGCATGCCTGCAGGTCGACTCGCAGTGCAGCGTGACCCG |
| Ubi1-R | ATTGATTTCCTGGGATCCAT AAGGCCTTTGCAGAAGTAACACCAA |
| AtALA1-F (+Ubi1, *Stu* I) | TTGGTGTTACTTCTGCAAAGGCCTTATGGATCCCAGGAAATCAATTG |
| AtALA1-R (+Ubi1, *Kpn* I） | TCGGGGAAATTCGAGCTCGGTACCTCATCTCCGTGGAGGATCCTG |
| AtALA1-F | ATGGATCCCAGGAAATCAATTG |
| AtALA1-R | AGCCAACCTATTGTTCTCAACTCTA |

**Supplementary Table 2. Plant height, hundred-kernel weight and seed diameter of T3 transgenic maize**

| Lines | Plant height (cm) | Hundred kernel weight (g) | Seed diameter (cm) |
| --- | --- | --- | --- |
| WT | 135.60 ± 18.70 | 19.69 ± 0.18 | 0.85 ± 0.025 |
| # 4 | 139.89 ± 11.08 | 20.46 ± 0.55 | 0.83 ± 0.008 |
| # 13 | 128.02 ± 22.36 | 19.50 ± 0.41 | 0.84 ± 0.017 |
| # 15 | 137.69 ± 18.52 | 19.38 ± 0.47 | 0.85 ± 0.013 |

**Supplementary Table 3. Vectors used in this study**

| **Vectors** | **Purpose** |
| --- | --- |
| proAtALA1::EGFP-AtALA1-Nos | Subcellular localization; mutant complementation |
| proAtALA1::AtALA7-eGFP-Nos |  |
| 35S-RFP-AtALA1-Nos | Subcellular localization |
| 35S-AtALA7-RFP-Nos |  |
| 35S-Pip2a-RFP-Nos |  |
| 35S-γ-Tip-RFP-Nos |  |
| 35S-RFP-RabA1d-Nos |  |
| 35S-RFP-RabF2a-Nos |  |
| 35S-AtALA1-Nos | Genetic transformation |
| 35S-AtALA7-Nos |  |
| Ubi1-AtALA1-Nos |  |
